# Deep learning-based prediction of the retinal structural alterations after epiretinal membrane surgery

**DOI:** 10.1038/s41598-023-46063-6

**Published:** 2023-11-06

**Authors:** Joseph Kim, Hee Seung Chin

**Affiliations:** 1 Department of Ophthalmology, Inha University School of Medicine, Incheon, Republic of Korea; 2 Retina Division, Nune Eye Hospital, Seoul, Republic of Korea

**Keywords:** Eye diseases, Retinal diseases

## Abstract

To generate and evaluate synthesized postoperative OCT images of epiretinal membrane (ERM) based on preoperative OCT images using deep learning methodology. This study included a total 500 pairs of preoperative and postoperative optical coherence tomography (OCT) images for training a neural network. 60 preoperative OCT images were used to test the neural networks performance, and the corresponding postoperative OCT images were used to evaluate the synthesized images in terms of structural similarity index measure (SSIM). The SSIM was used to quantify how similar the synthesized postoperative OCT image was to the actual postoperative OCT image. The Pix2Pix GAN model was used to generate synthesized postoperative OCT images. Total 60 synthesized OCT images were generated with training values at 800 epochs. The mean SSIM of synthesized postoperative OCT to the actual postoperative OCT was 0.913. Pix2Pix GAN model has a possibility to generate predictive postoperative OCT images following ERM removal surgery.

## Introduction

Epiretinal membrane (ERM) is an avascular fibrocellular proliferative layer that develops on the surface of the inner retina. In the previous studies of ERM, the prevalence has been reported to be 6.0–11.8% for westerners, and 2.2–7.9% for Asians, and ERM is more common in people over 50 years of age^1–6^. When ERM causes structural changes on the macula, it causes symptoms such as monocular diplopia, metamorphopsia, or reduced visual acuity. Surgical ERM removal is a well-established treatment of choice to improve visual quality in symptomatic patients with ERM^7–9^.

Recently, optical coherence tomography (OCT) has been used to quantify the structural changes of retina^10^. Many studies have reported the correlations between postoperative visual improvement and retinal microstructure in OCT images in ERM patients. These include preoperative photoreceptor integrity^11,12^, presence of ectopic inner foveal layer^13^, the ratio of foveal outer nuclear layer (ONL) thickness to the juxtafoveal ONL plus outer plexiform layer thickness^14^, preoperative inner nuclear layer thickness^15,16^, inner retinal irregularity index^17^, photoreceptor outer segment length^18^, central foveal thickness and central inner retinal layer thickness^19^.

Regarding postoperative OCT parameters related to visual prognosis in ERM, Kim et al. showed a significant correlation between final BCVA and early postoperative central macular thickness (CMT). The CMT at 1 month after surgery was more related to the final visual outcome than CMT at 3 and 6 months^20^. Romano et al. reported that an increase in retinal nerve fiber layer thickness at 1 month and a decrease in ONL thickness at 3 and 6 months follow-up were associated with a good final BCVA^21^. Mahmoudzadeh et al. demonstrated that postoperative inner retinal microcysts or ellipsoid zone disruption at 3 month was related to poor final visual outcome^22^.

However, despite successful ERM surgery, some patients are dissatisfied with their postoperative visual acuity. This may be because the patient’s expectations are too high or the patient’s surgical outcome is really lower than the surgeon’s empirical expectations.

Many clinicians’ efforts and their studies related to the various prognostic factors of ERM surgery have enabled surgeons to explain the expected visual outcome of surgery with greater predictive power to the patients before the surgery. However, since no one can time travel, the postoperative OCT biomarkers cannot be used to predict surgical outcome in advance of the surgery. If the postoperative OCT image can be specified in details before surgery, it is expected to help improve the prognosis prediction of the surgery.

Recently, with the rapid growth of researches on artificial intelligence, efforts are being made to use it in various ways in the field of medicine^23^. In particularly, deep neural network has made a remarkable progression. Generative adversarial network (GAN), a representative deep neural network, was firstly developed by Ian Goodfellow^24,25^. GAN is a model that generates a variety of realistic fake images originated from the data distribution in datasets. GAN consists of two learning models. One is a model that generates a false synthetic image, called a generator (G), and it is trained to generate a fake image as real as possible. The other model is called a discriminator (D) and it is trained to determine whether the synthesized image generated by G has been manipulated. If these two models are trained as if they are competing with each other, ideally G can generate realistic fake images^25^. GAN has been widely used for medical image processing, especially in the field of radiology. Previous studies have shown that GAN can be used for image reconstruction, denoising, data augmentation, domain transfer between modalities, segmentation, classification and detection etc.^24,26,27^. Shitrit et al. introduced a method that can secure good quality images while reducing MRI scan time by generating estimated images using GAN^28^. Wang et al. have published several papers using GAN which include a study on segmenting the basal membrane in histopathology images of microinvasive cervix carcinoma, a study on removing metal artifacts from CT images, and converting low-quality PET brain images into high-quality images^29–31^. Ben-Cohen et al. demonstrated domain translation, which converts a CT image into a PET image^32^. In a study on liver lesion classification, Frid-Adar et al showed that synthetic data augmentation using GAN can improve the performance of the classifier^33^.

In the field of ophthalmology, several studies have been attempted to predict intervention results by generating predictive post interventional images using GAN^34–36^.

Therefore, the author would like to confirm whether it is possible to predict the postoperative structural changes of the patient in ERM using deep learning methodology known as GAN. The results of this study are expected to serve as a stepping stone for further researches to analyze the relationship between the predicted retinal structure and visual prognosis of ERM surgery in the future.

## Materials and methods

This study adhered to the tenets of the Declaration of Helsinki and received approval from the Nune Eye Hospital Institutional Review Board/Ethics Committee (N-2304-001-999). The requirement for informed consent was waived by the Nune Eye Hospital Institutional Review Board/Ethics Committee because of the retrospective nature of this study.

### Study design and dataset creation

The author reviewed the medical records of patients who were diagnosed with idiopathic ERM and underwent ERM removal surgery at Nune Eye Hospital in Seoul, South Korea from January 1, 2011 to December 31, 2022. Patients with secondary ERM, myopia of more than 26mm of axial length, presence of any other retinal disease were excluded.

All surgeries were performed by 12 retinal surgeons, and 25-gauge pars plana vitrectomy (PPV) was performed under retrobulbar anesthesia using the Constellation Vision System (Alcon, Fort Worth, TX, USA) and a high refractive index contact lens. After core vitrectomy, triamcinolone acetonide or 0.25% indocyanine green (ICG) was used to visualize the ERM, followed by removal of the epiretinal and internal limiting membranes using intraocular forceps. According to the doctor's decision, phacoemulsification and intraocular lens implantation were performed together with PPV for patients who needed cataract surgery.

A pair of horizontal preoperative and postoperative OCT (Spectralis, Heidelberg Engineering, Heidelberg, Germany) scan image of each patient’s macular center was obtained. The authors manually selected images passing through the fovea in the OCT volume scan. The author used the OCT image examined at 1 month after surgery as the postoperative OCT image. Pre and postoperative OCT image pairs were divided into two sets; a training dataset for model training and a test dataset for model evaluation.

**Figure 1:**
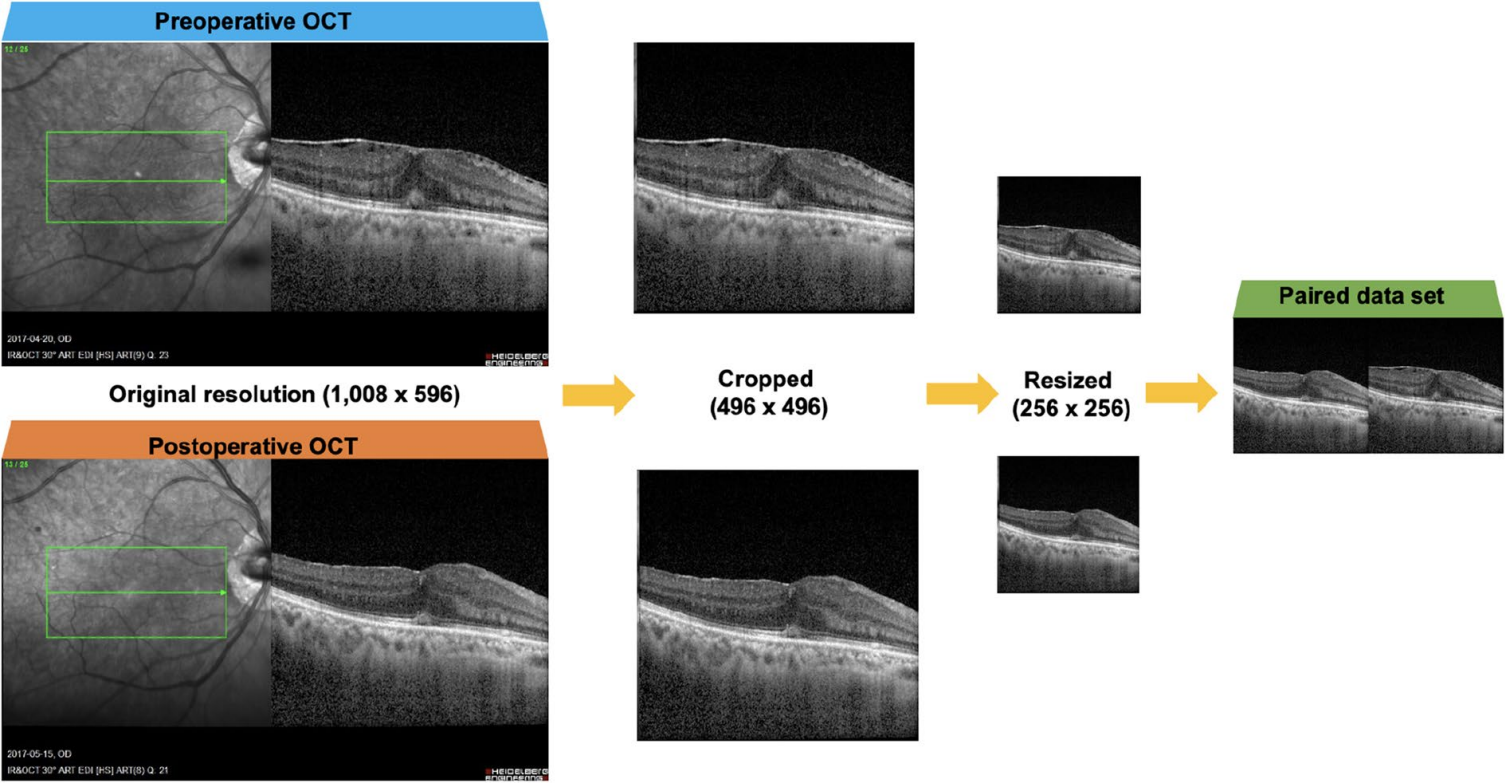
Conceptual illustration of creating a paired dataset combining pre and postoperative OCT images.

All Macular OCT images were cropped from their original resolution of 1008 × 596 pixels to 496 × 496 pixels in order to exclude en face image and resized to 256 × 256 pixels for deep learning (Fig. 1, Supplementary Fig. 1).

### Synthesizing postoperative OCT image

#### Conditional generative adversarial network

Conditional generative adversarial network (cGAN) is a conditional version of GAN. Since GAN is an unconditioned generative model, realistic images are randomly generated and there is no option to control the synthesized image. Mirza et al. proposed a cGAN in which conditional data *y* can be added to G and D^37^.

The objective function of cGAN is defined as follows:

1 $$\underset{G}{\mathrm{min}}\underset{D}{\mathrm{max}}V(D,G)= {\mathbb{E}}_{x\sim {p}_{\mathit{data}}(x)}\left[\mathrm{log}D\left(x|y\right)\right]+{\mathbb{E}}_{z\sim {p}_{z}(z) }\left[\mathrm{log}\left(1-D(G\left(z|y\right))\right)\right],$$

*D*: discriminator, *G*: generator, *P*_data_: probability distribution of dataset, *P*_z_: noise distribution to be used to sample a random noise vector, *x*: image in dataset, *z*: random noise vector, *y*: a condition.

In cGAN, it can be modulated to generate images corresponding to a condition *y*, such as class labels.

#### Pix2Pix (image to image translation with cGAN)

In 2017, Isola et al. proposed Pix2Pix, a universal image to image translation with cGAN, that can effectively perform various image translation tasks without changing the framework to be fit to specific applications^38^.

In GAN, G is trained to learn a mapping to the actual data space from random noise. On the other hand, in cGAN, G is learning a mapping to the actual data space from random noise plus a specific condition. Meanwhile, in Pix2Pix, G is trained to learn a mapping from the input image, which is a condition, to the actual ground truth.

The objective function of Pix2Pix is defined as follows^38^:

2 $${G}^{*}= arg\underset{G}{\mathrm{min}}\underset{D}{\mathrm{max}}{\mathcal{L}}_{cGAN} (G,D) + \lambda {\mathcal{L}}_{L1}(G) ,$$

3 $${\mathcal{L}}_{\mathit{cGAN}} (G,D)= {\mathbb{E}}_{x,y}\left[\mathrm{log}D(x,y)\right]+{\mathbb{E}}_{x,z }\left[\mathrm{log}\left(1-D(x,G(x,z)\right)\right] ,$$

4 $${\mathcal{L}}_{L1} (G)= {\mathbb{E}}_{x,y,z}\left[\parallel y-G(x,z){\parallel }_{1}\right] ,$$

*D*: discriminator, *G*: generator, *x*: image in dataset, *z*: random noise vector, *y*: ground truth image, *L*: loss function.

Pix2Pix generates data that seems like real, such as the equation of (3). As defined in the equation of (4), the generated image is synthesized such that the difference from the actual ground truth is minimized. Therefore, Pix2Pix generates specific deterministic images corresponding to the ground truth images, rather than generating a variety of realistic images that are characteristic of previous GAN or cGAN.

We use Pix2Pix model to generate predictive postoperative OCT images. The Pix2Pix network architecture was implemented using Python (V.3.6) programming language in PyCharm with Anaconda virtual environment as described in the original article^38^. The G architectures are as follows.

Encoder: C64-C128-C256-C512-C512-C512-C512-C512,

U-Net decoder: CD512-CD1024-CD1024-C1024-C1024-C512-C256-C128,

The D architectures are as follows:

C64-C128-C256-C512,

Ck: a Convolution-BatchNorm-ReLU layer with k filters. CDk: a Convolution-BatchNorm- Dropout-ReLU layer with a dropout rate of 50%. Convolutions: 4 × 4 spatial filters applied with stride 2. Convolutions in the encoder, and in the discriminator, downsample by a factor of 2, whereas in the decoder they upsample by a factor of 2. All ReLUs in the encoder and the discriminator are leaky, with slope 0.2, while ReLUs in the decoder are not leaky^38^.

**Figure 2:**
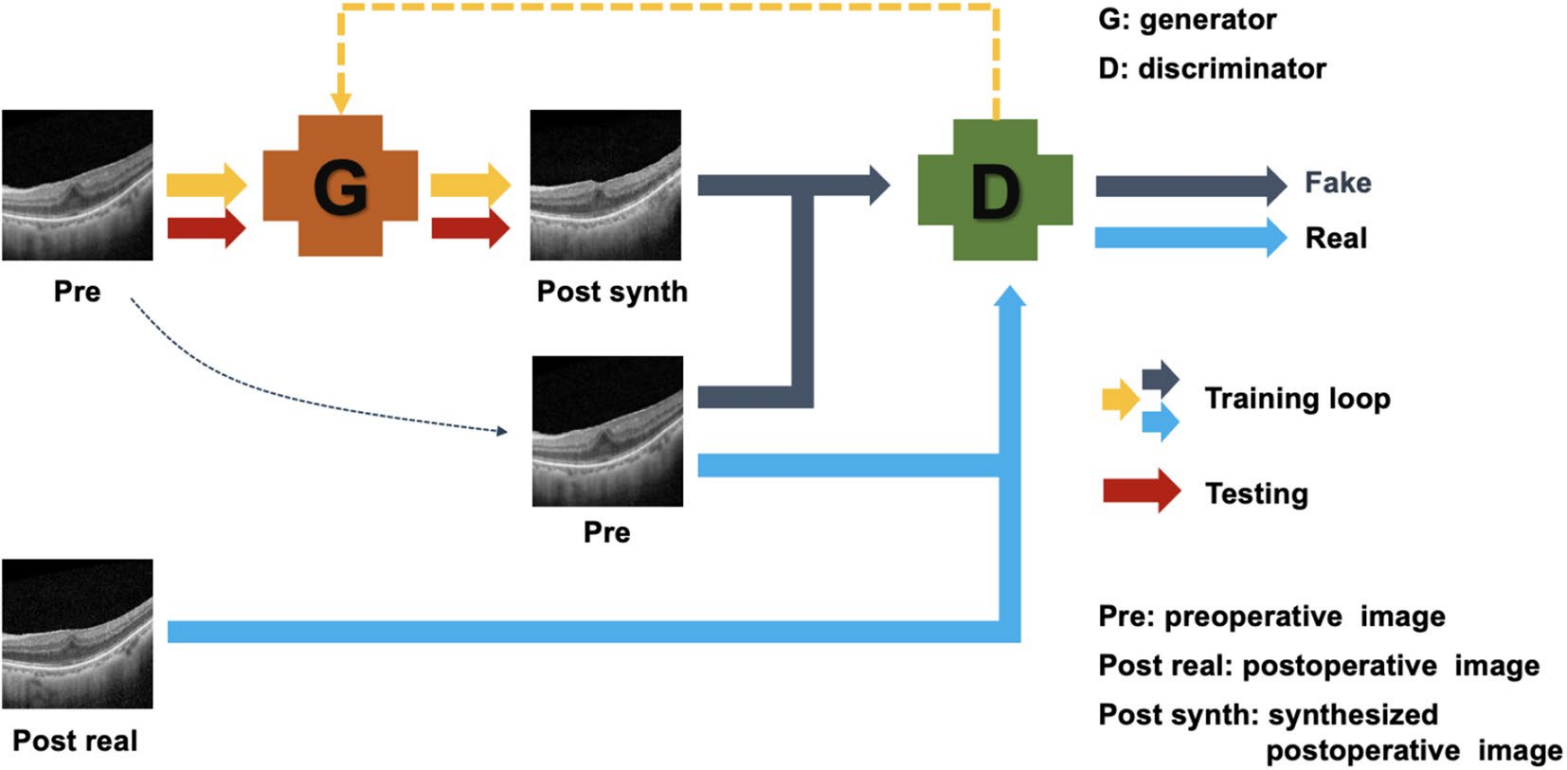
A conceptual drawing of the Pix2Pix model used in our study for synthesizing predictive postoperative OCT image.

In Pix2Pix, G was trained to produce a synthesized postoperative OCT image with preoperative OCT image as an input. D was trained to determine whether the synthesized postoperative image was a real postoperative OCT or a fake image created by the G (Fig. 2).

All training and testing were performed using Google Colaboratory, which can run a .py file.

### Evaluation of synthesized postoperative OCT image

#### Structural similarity index measure

The structural similarity index measure (SSIM) was used to evaluate how similar the synthesized postoperative OCT image was to the real postoperative OCT image. The SSIM is a well-known image quality assessment metrics to measure the similarity between two given images based on statistical changes in image luminance, contrast and structure. The value of SSIM is between 0 and 1. the closer to 1, the more similar the two images are^39^.

#### Image binarization using adaptive thresholding

The author binarized images to minimize the effects of differences in luminance and contrast of the corresponding retinal layers between the synthesized image and the real OCT image when SSIM evaluation. Binarization is to classify image pixels as either black and white. The author binarized the images using an adaptive binarization method known to provide qualified segmentation despite strong illumination changes in the images^40^.

#### Region of interest

**Figure 3:**
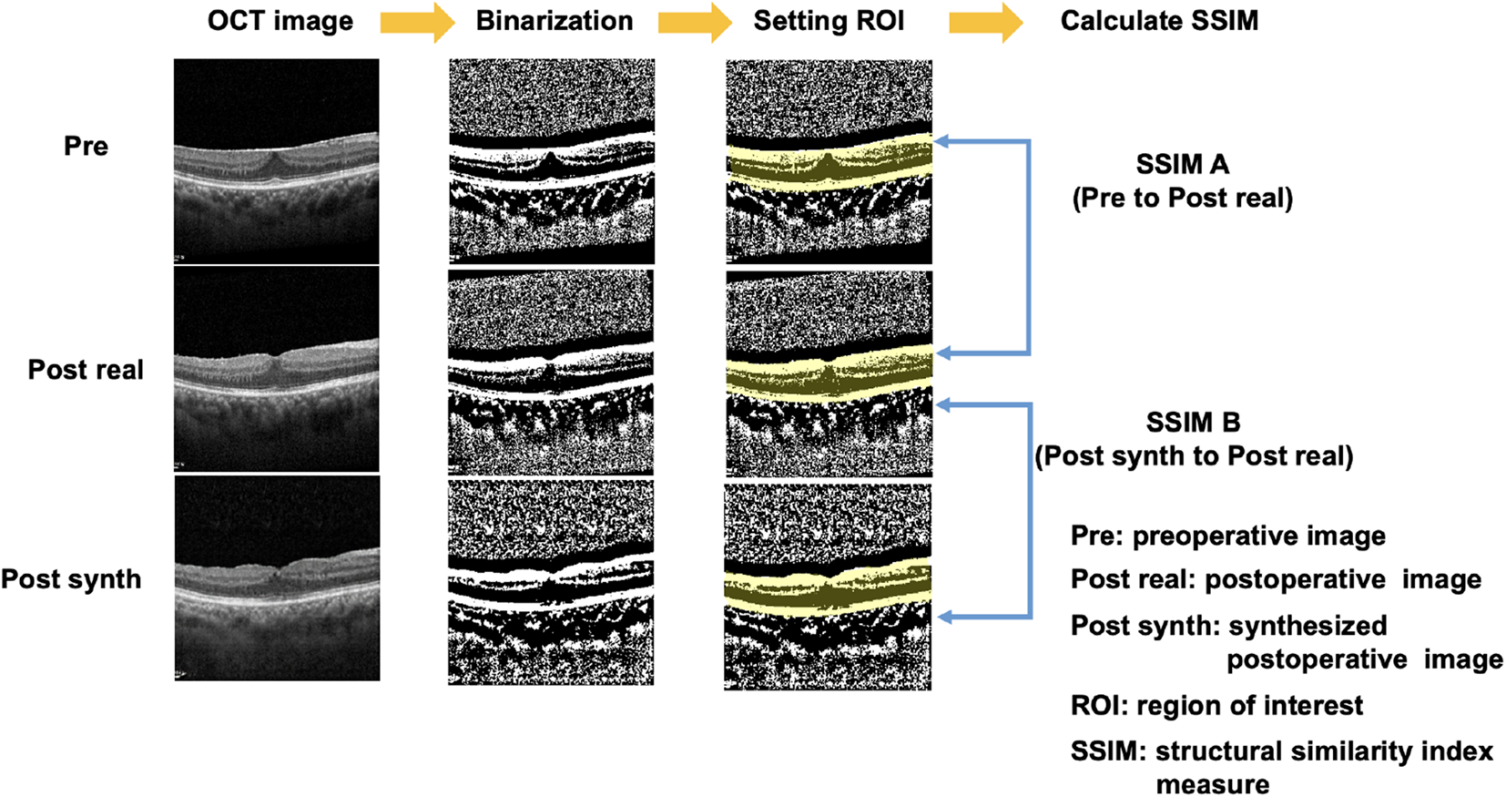
A schematic workflow for calculating structural similarity index measure.

We set the region of interest (ROI) from the innermost surface of retina to the boundary of choroid on binarized images using the AssistedFreehand function (hand-drawn ROI, where the line drawn automatically follows edges in the underlying image) in MATLAB and then calculated the each SSIM. The author named the SSIM of the preoperative image to the postoperative image as SSIM A, and the SSIM of the synthesized image to the postoperative image as SSIM B. All these assessments were performed using MATLAB R2021a software (Fig. 3).

### Statistical analysis

Statistical analysis was performed using the R software. To compare baseline characteristics between each dataset, the Wilcoxon rank sum test for the age, the chi-square test for the sex and the combined cataract surgery, the Cochran-Armitage test for ERM stage, and the *t* test for the axial length were used. *P* < 0.05 was considered statistically significant.

## Results

### Baseline characteristics

The training dataset consisted of a total of five hundred pairs of OCT images and the test dataset consisted of sixty pairs of OCTs. Therefore, total sixty synthesized postoperative OCT images were generated based on the preoperative OCT images in the test dataset. In the training dataset, the mean age of patients was 63.70 ± 8.00 years, and 59.8% were female. In the test dataset, the mean age of patients was 64.27 ± 6.39 years, and 70.0% were female. All ERM stages were stage 2 or higher in both datasets. A total of 313 (62.6%) cases in the training data set and 40 (66.7%) cases in the test data set had received combined cataract surgery with PPV. Axial length was 23.35 ± 0.75 in the training dataset and 23.42 ± 0.89 in the testing dataset (Table 1).

**Table 1:** Baseline characteristics.

	Training dataset	Testing dataset	*p* value
Age, years	63.70 ± 8.00	64.27 ± 6.39	0.767
Female (%)	299 (59.8)	42 (70.0)	0.187
ERM stage			0.877
Stage 1 (%)	0 (0)	0 (0)	
Stage 2 (%)	188 (37.6)	20 (33.3)	
Stage 3 (%)	259 (51.8)	38 (61.7)	
Stage 4 (%)	53 (10.6)	3 (5.0)	
Combined cataract surgery (%)	313 (62.6)	40 (66.7)	0.678
Axial length, mm	23.35 ± 0.75	23.42 ± 0.89	0.933

The Wilcoxon rank sum test for the age, the chi-square test for the sex and the combined cataract surgery, the Cochran-Armitage test for ERM stage, and the *t* test for the axial length were used.

### Pix2Pix model training and synthesized OCT images from the test dataset

**Figure 4:**
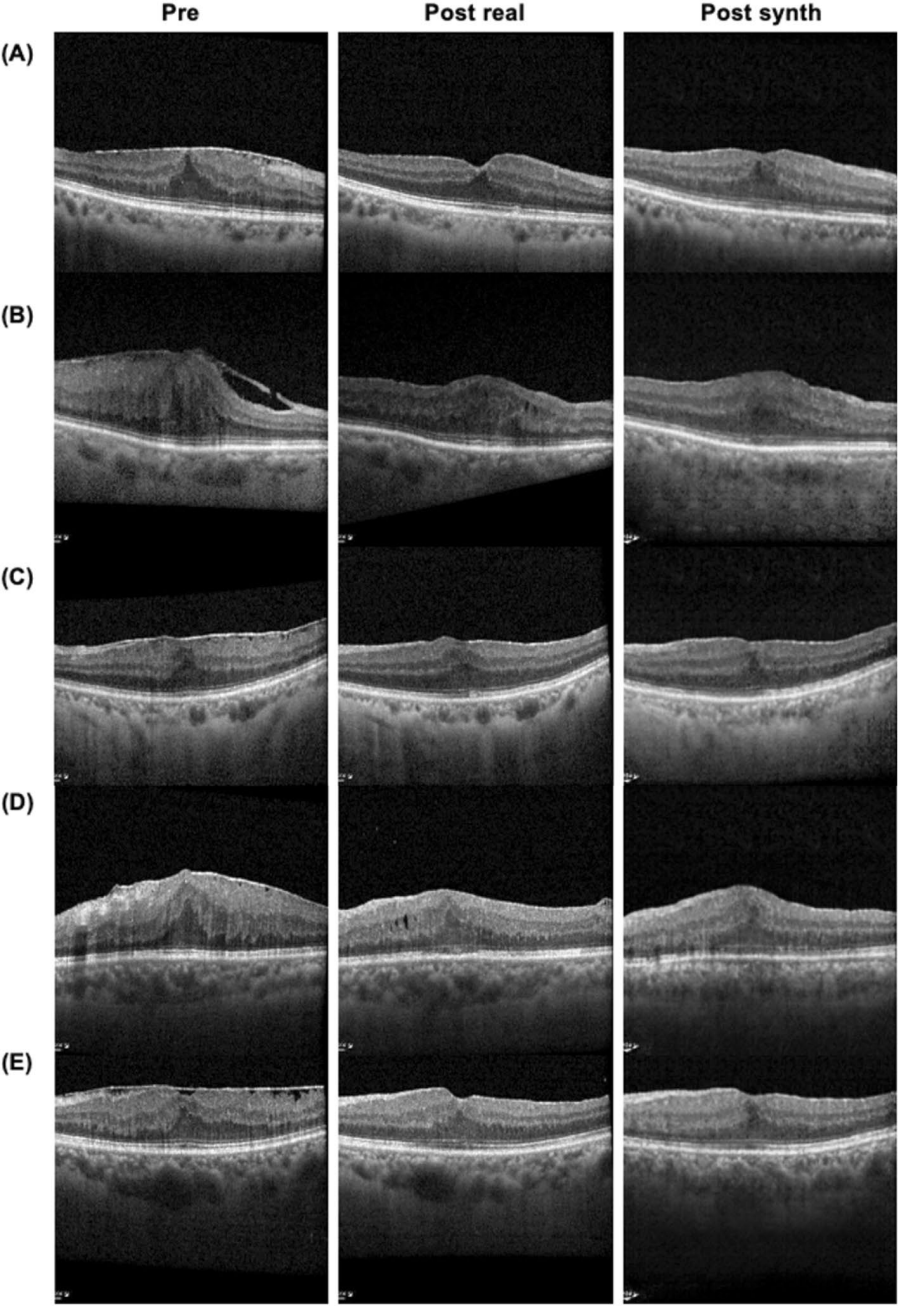
Representative synthesized postoperative OCT images. *Pre* preoperative OCT, *Post real* an actual postoperative OCT, *Post synth* synthesized postoperative OCT.

The model was trained with 500 pairs of OCT images for 800 epochs, batch size 10, Adam optimizer, learning rate of 2 × 10^–4^, with random jitter and mirroring. The synthesized OCT images were generated using training values for 800 epochs. A total of sixty synthesized postoperative OCT images were generated (Fig. 4, Supplementary Fig. 2).

### Similarity evaluation of synthesized OCT images

**Figure 5:**
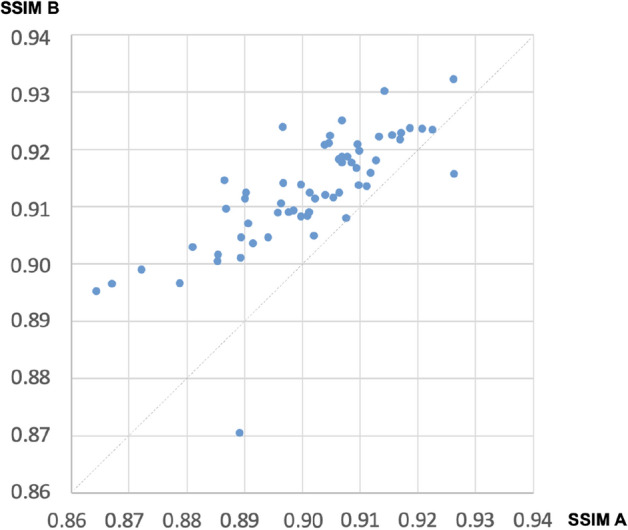
SSIM B versus SSIM A. *SSIM* structural similarity index measure, *SSIM A* SSIM for preoperative to real postoperative OCT, *SSIM B* SSIM for synthesized postoperative to real postoperative OCT.

**Figure 6:**
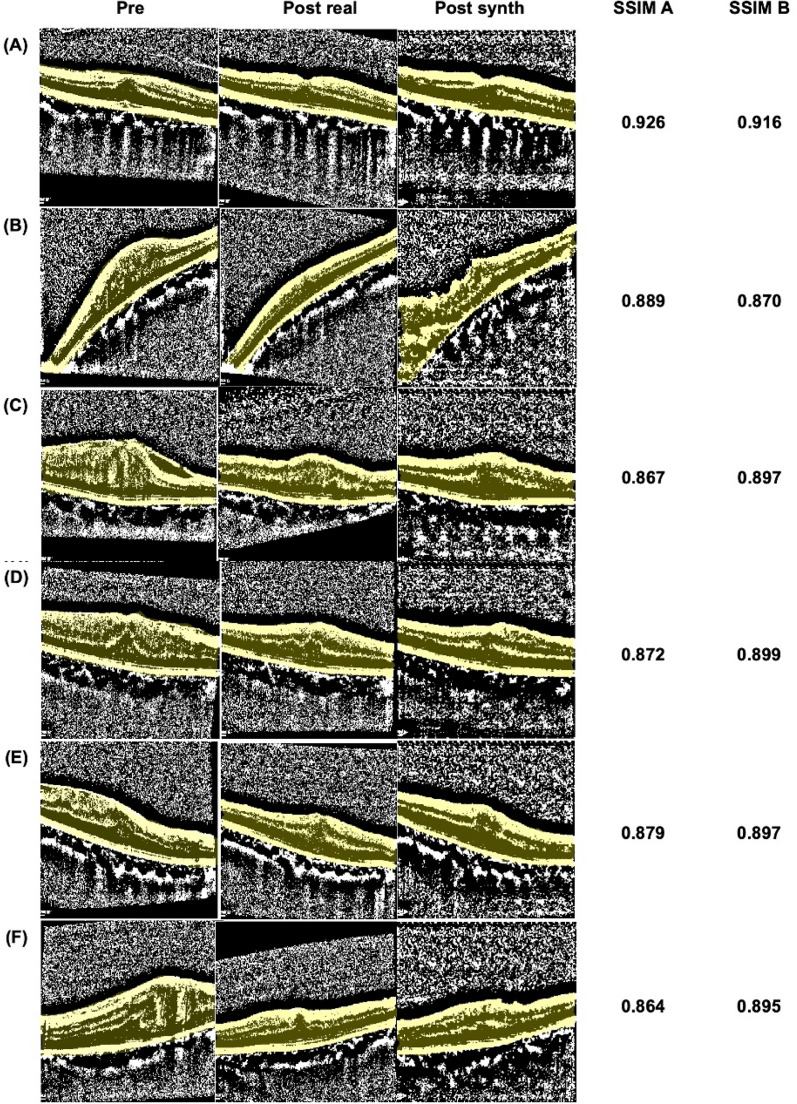
Representative images for SSIM A and SSIM B. *SSIM* structural similarity index measure, *SSIM A* SSIM for preoperative to real postoperative OCT, *SSIM B* SSIM for synthesized postoperative to real postoperative OCT, *Pre* preoperative OCT, *Post* *real* an actual postoperative OCT, *Post* *synth* synthesized postoperative OCT.

The SSIM A and B were calculated for sixty image pairs, respectively. For 58 out of 60 images, the SSIM of the synthesized images was higher than that of the preoperative images (Fig. 5). The two images where SSIM B is lower than SSIM A are shown in (A) and (B) of Fig. 6.

## Discussion

In this study, the author generated and evaluated synthesized postoperative OCT images to predict structural changes that occur after ERM removal surgery.

### Similarity evaluation

The similarity of OCT images between synthesized and real postoperative was evaluated using SSIM. SSIM is one of the image quality assessment (IQA) methods.

Traditional method of IQA is subjective human evaluation to determine how good the quality of an image is compared to a reference image. This is usually too inconvenient, time-consuming and expensive to acquire reliable conclusions. To overcome these drawbacks, methods for conducting objective IQA have been developed. There are several popular IQAs. The most popular and widely used IQA is mean squared error, calculated by averaging the squared intensity differences between image pixels. But these kind of IQAs are not very well matched to the image quality perceived by human. SSIM was developed in an effort to evaluate image quality in the way that humans perceive images. It is based on the hypothesis that the human visual system is highly adapted with the perception of structural information. SSIM is expressed in luminance, contrast, and structure information. After grouping the pixels of the image in small window units and calculating each SSIM, the mean SSIM is obtained through the average of the values, and a value between [0, 1] is presented as an evaluation index. The closer the value is from 0 to 1, the more similar the images are. If 1, both images are identical^39,41^.

Many researches on image-to-image translation are being actively conducted in the field of medicine. Several previous studies using SSIM as an image quality assessment presented various SSIM values ranging from 0.570 to 0.920^41–46^.

Our results show 0.913 of mean SSIM value for the synthesized images to real postoperative images. The SSIM value of each synthesized image is higher than SSIM of the preoperative OCT to real postoperative image except for two cases (Figs. 5, 6). Of the two cases, one was not synthesized properly, and the other case had the mildest preoperative retinal distortion (Fig. 6A,B). Therefore, it can be reasonably concluded that the author generated predictive postoperative OCT images by using deep learning methodology known as Pix2Pix. This implies that a kind of cGAN methodology has the potential to be used to synthesize predictive postoperative OCT images of ERM patients.

There were five images with SSIM B values less than 0.9 (Fig. 6B–F). Three of these were ERM stage 4 and the other two were ERM stage 3. Among the test dataset images, there were a total of three of ERM stage 4, and all of them have relatively low SSIM values. Therefore, the more deformed the retina, the more difficult it was to predict postoperative OCT images in our model. Other characteristics of them, such as axial length and degree of myopia, were not different from the other patients. Meanwhile, the lowest SSIM value of 0.87044 for image shown in (B) of Fig. 6 seems to be too high considering that image (B) was not generated satisfactorily. Therefore, there remains room for complementing image quality assessment to evaluate the synthesized images.

### Post-intervention predictions using GAN in the field of ophthalmology

Three studies reported post therapeutic predictions using GAN in the field of ophthalmology^47^. To our knowledge, Yoo et al. proposed a concept to generate the post therapeutic predictive images using GAN at the first time^34^. He presented a synthesized postoperative appearance after orbital decompression surgery. Yoo et al. trained a GAN model with109 image pairs of pre and postoperative facial images of patient who underwent orbital decompression surgery due to thyroid-associated ophthalmopathy with data augmentation. They explained that the predicted postoperative images were similar to the actual postoperative images and that images generated using GANs would help patients predict the impact of orbital decompression surgery in advance. Although the resolution of the synthesized image was relatively low (64 × 64 pixels), the result showed the applicability of GAN to oculoplastic surgery. The other two studies were on treatment prediction for patients with exudative age-related macular degeneration (ARMD). Liu et al. trained a GAN model with 479 image pairs of pre and post therapeutic OCT images (256 × 256 pixels) of patient who received intravitreal anti vascular endothelial growth factor (VEFG) injection, and then the performance of the GAN model was evaluated using 50 OCT image pairs in the test sets. Liu et al. showed that 92% of the synthesized images had acceptable quality with an 85% accuracy to predict the post treatment macular status in their study. They insisted that GAN has great potential to synthesize post interventional OCT images with good quality and high accuracy in ARMD treatment^35^. Lee et al. proposed a conditional GAN with a multi-channel input for the post interventional prediction for anti-VEGF injection treatment. They trained the GAN model through data augmentation using 723 OCT image pairs (256 × 256 pixels) combined with fluorescein angiography or indocyanine green angiography, and then the performance of the GAN was evaluated using 150 OCT image pairs in the test sets. They showed that a conditional GAN was able to generate post therapeutic OCT images using baseline OCT, fluorescein angiography and indocyanine green angiography images in ARMD treatment^36^. However, these studies had several limitations in terms of short-term follow-up periods, selection bias of excluding poorly synthesized images.

### Limitations

Our study also has limitations that were suggested in the previous studies.

First, we had to downsize the OCT images to train and test the model. This inherently has a possibility of losing the original information for images. The next limitation is short term follow-up period. The shape of the retina gradually changes over time after surgery. Therefore, the longer the follow-up period, the more difficult it is to predict the shape of the retina after ERM surgery. Therefore, a subsequent study on long term follow-up period is needed to confirm the performance of the model. Third, the author evaluated the quality of the synthesized sample images by regularly saving network weights and sample images during training to determine when to stop training. Since the optimal performance point of the generator was not evaluated with the validation dataset, it cannot be free from the possibility of overfitting. Fourth, alignment issues in paired images may affect the accuracy of SSIM. Finally, this study included multiple surgeons (a total of 12 retina specialist) who had different surgical experience. Depending on the surgeon, the extent of removal of the epiretinal membrane and the internal limiting membrane could be different, and the degree of microtrauma to the retina during surgery might not be identical. In other words, each surgeons have different skills. Therefore, this kind of surgeon’s factor has the possibility to affect the structural recovery as a confounding factor.

## Conclusion

In conclusion, the author generated postoperative OCT images and demonstrated that Pix2Pix GAN model has the possibility to generate predictive postoperative OCT images following ERM removal surgery. This result is expected to serve as a stepping stone for further researches to analyze the relationship between the predicted retinal structure and visual prognosis of ERM surgery. The authors anticipate that this study may also stimulate further researches on the application of GANs in the field of interventional or therapeutic image prediction in ophthalmology.

### Supplementary Information

Supplementary Figure 1.

Supplementary Figure 2.

## Data Availability

All relevant data that support the findings of this work are available in this manuscript and supplementary material. The raw datasets are available from the corresponding author upon reasonable request only.
