# Author Correction: Deep learning-based prediction of the retinal structural alterations after epiretinal membrane surgery

**DOI:** 10.1038/s41598-023-47940-w

**Published:** 2023-11-23

**Authors:** Joseph Kim, Hee Seung Chin

**Affiliations:** 1 Department of Ophthalmology, Inha University School of Medicine, Incheon, Republic of Korea; 2 Retina Division, Nune Eye Hospital, Seoul, Republic of Korea

Correction to: *Scientific Reports*, 10.1038/s41598-023-46063-6, published on 06 November 2023.

The original version of this Article erroneously included the Acknowledgements. It has consequently been removed.

The original Article has been corrected.

