# Supplementary material for: Deep learning-based prediction of the retinal structural alterations after epiretinal membrane surgery
Source: Sci Rep. 2023 Nov 6;13:19275. doi: 10.1038/s41598-023-46063-6 (PMC10630279; doi:10.1038/s41598-023-46063-6)
Supplement: Supplementary file 1 — Supplementary Figure 1. [file 41598_2023_46063_MOESM1_ESM.pdf]

Sample of paired images

(1) **Postoperative Image** **Preoperative Image**

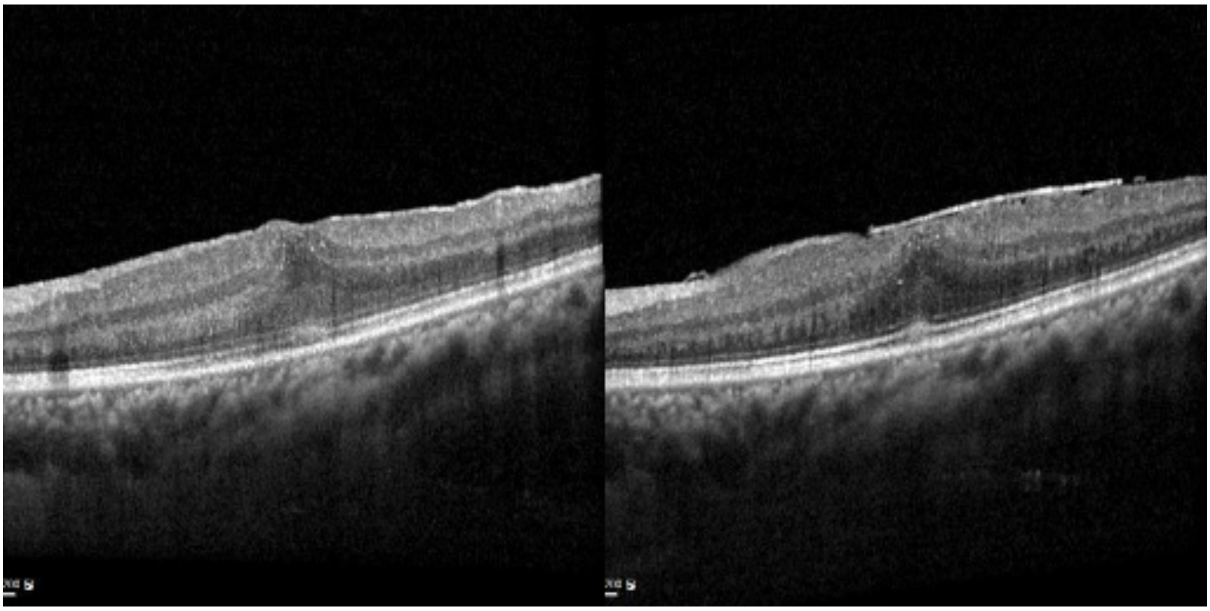

(2) **Postoperative Image** **Preoperative Image**

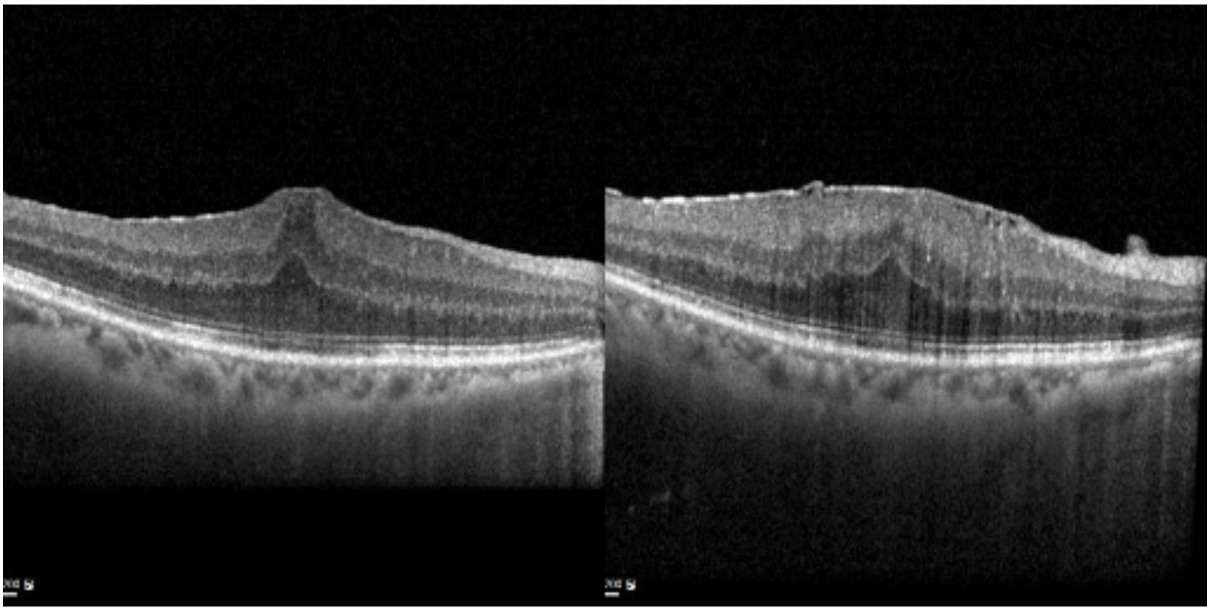

Supplementary Figure 1. Sample of paired images in training data set.
