# Supplementary material for: Deep learning-based prediction of the retinal structural alterations after epiretinal membrane surgery
Source: Sci Rep. 2023 Nov 6;13:19275. doi: 10.1038/s41598-023-46063-6 (PMC10630279; doi:10.1038/s41598-023-46063-6)
Supplement: Supplementary file 2 — Supplementary Figure 2. [file 41598_2023_46063_MOESM2_ESM.pdf]

Sixty synthesized postoperative OCT Images.

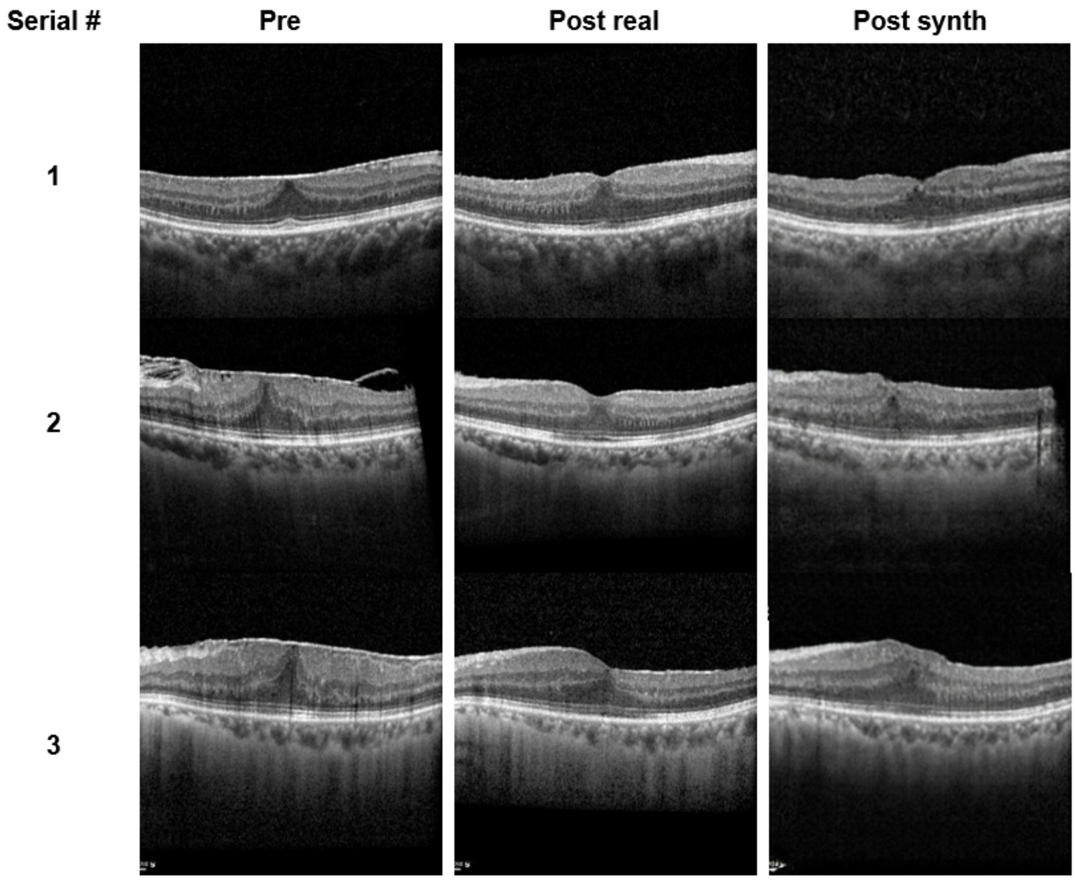

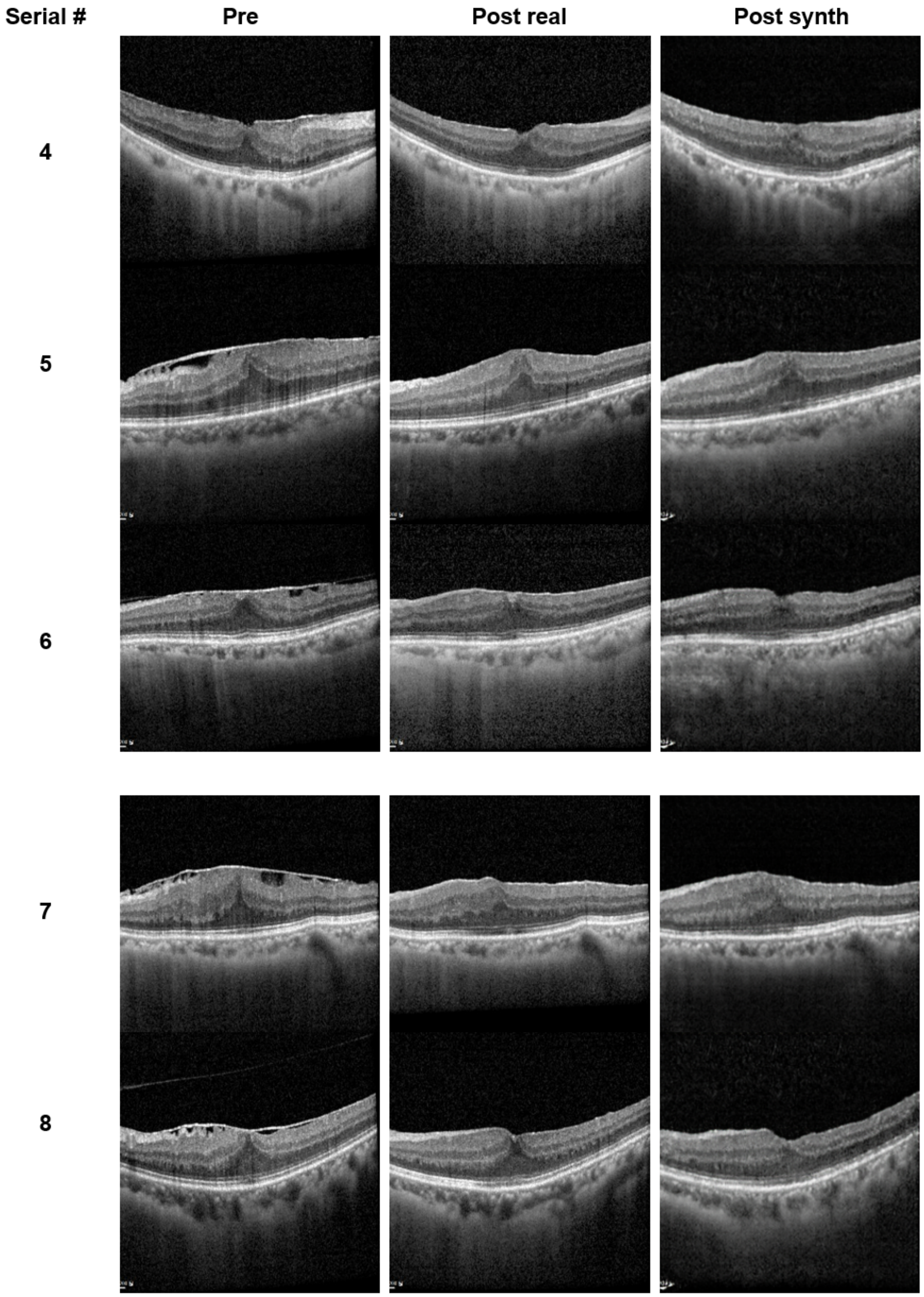
Serial #
Pre
Post real
Post synth
4
5
6
7
8

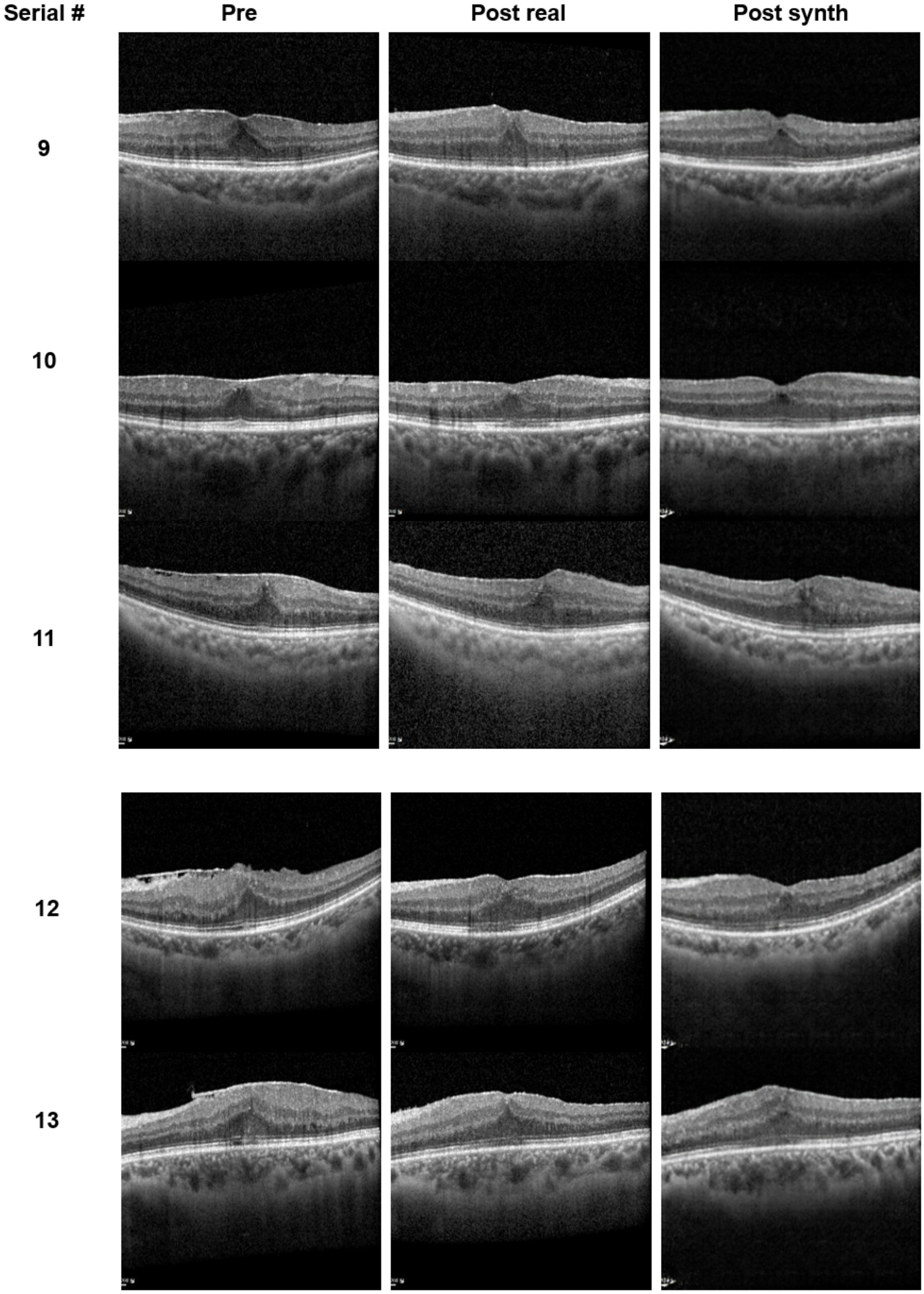
Serial #
Pre
Post real
Post synth
9
10
11
12
13

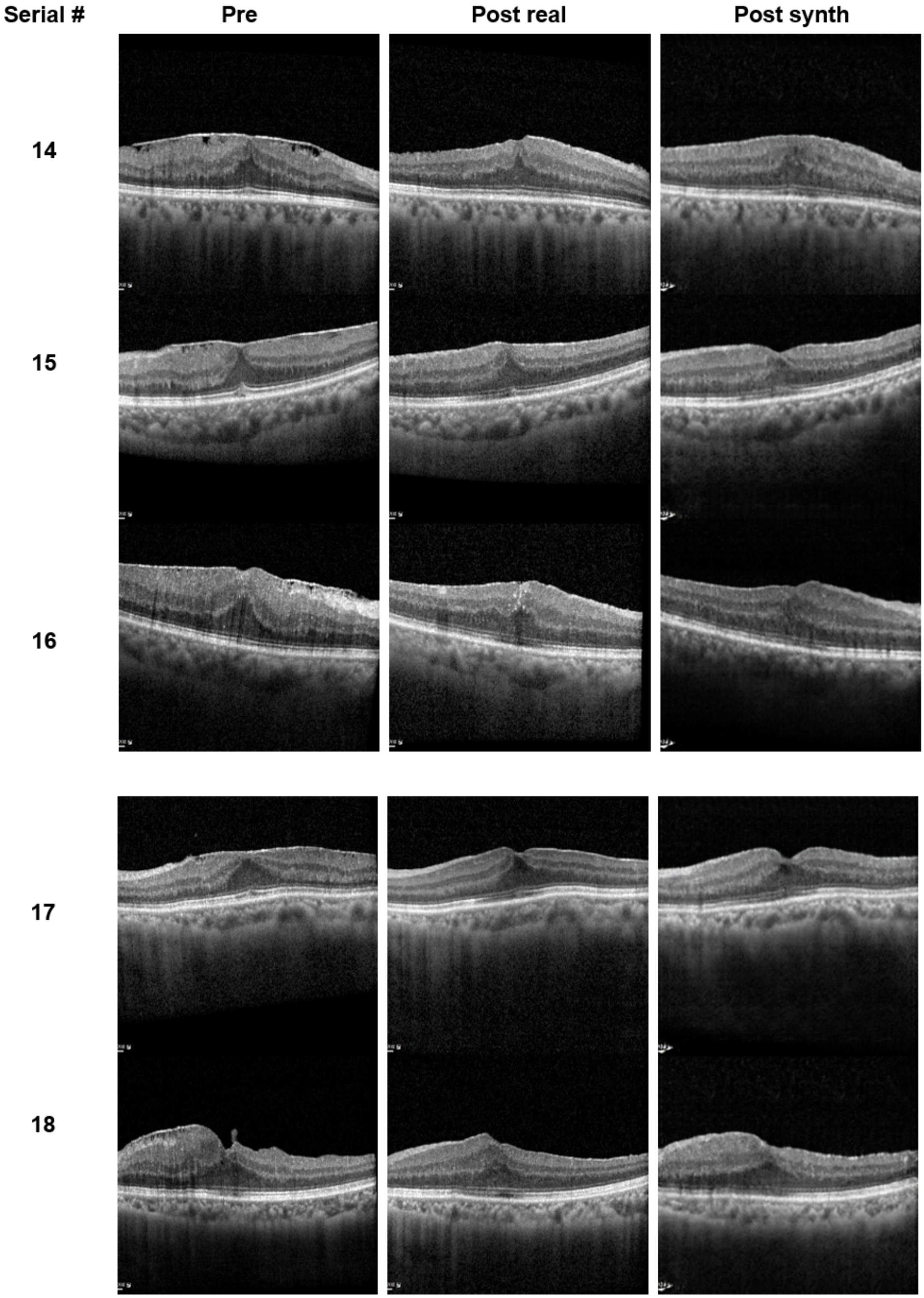
Serial #
Pre
Post real
Post synth
14
15
16
17
18

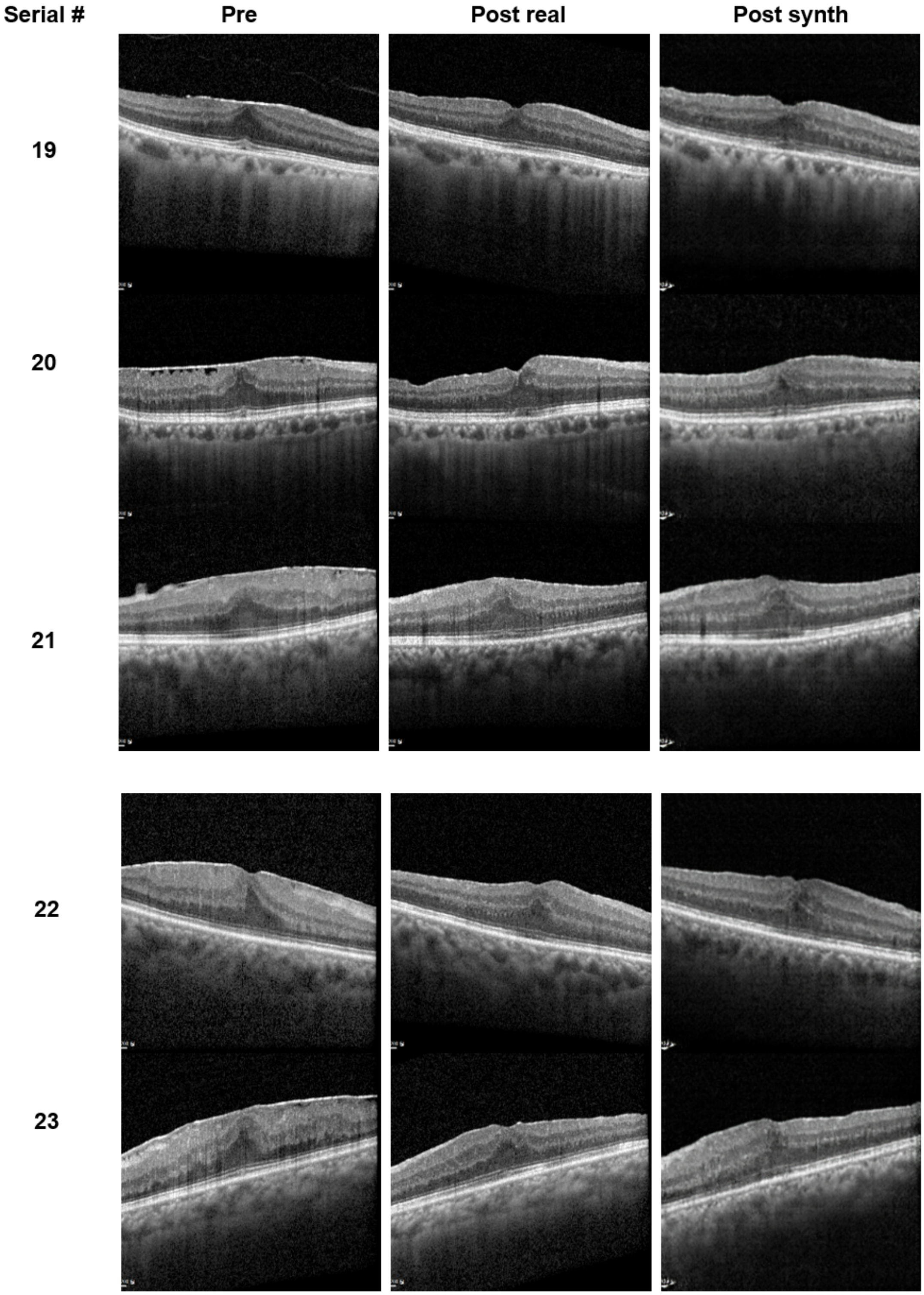

Serial #
Pre
Post real
Post synth
19
20
21
22
23

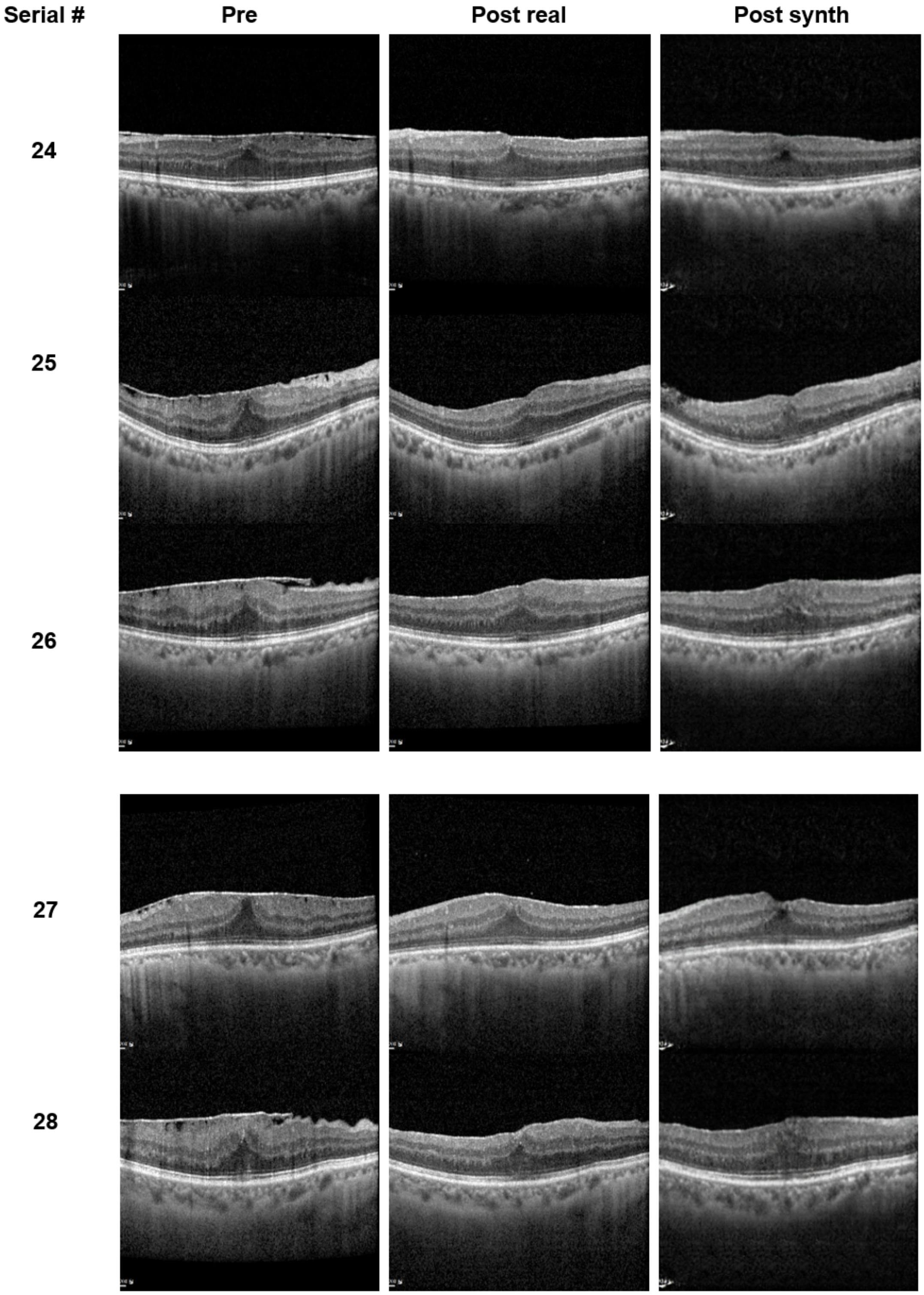
Serial #
Pre
Post real
Post synth
24
25
26
27
28

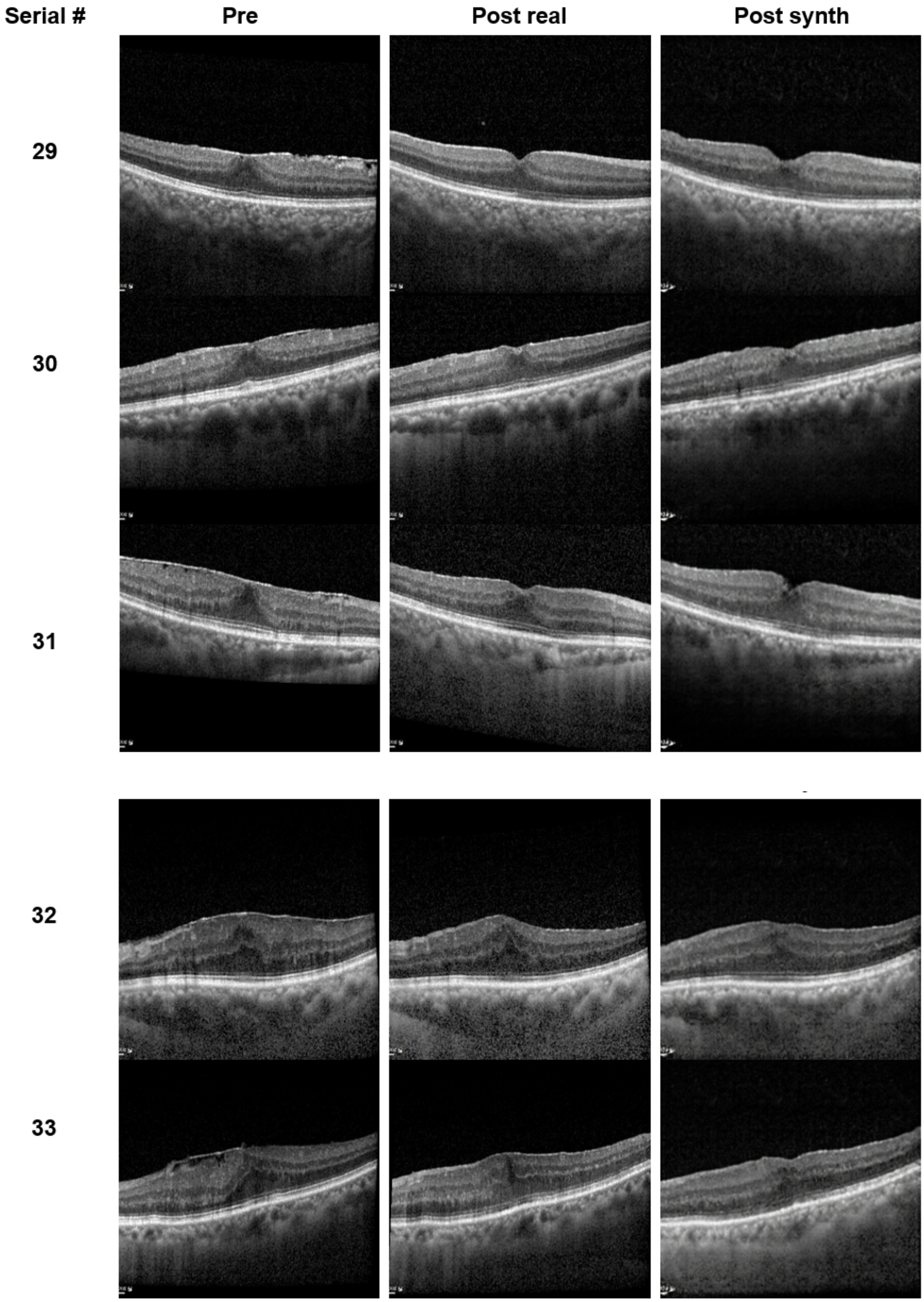
Serial #
Pre
Post real
Post synth
29
30
31
32
33

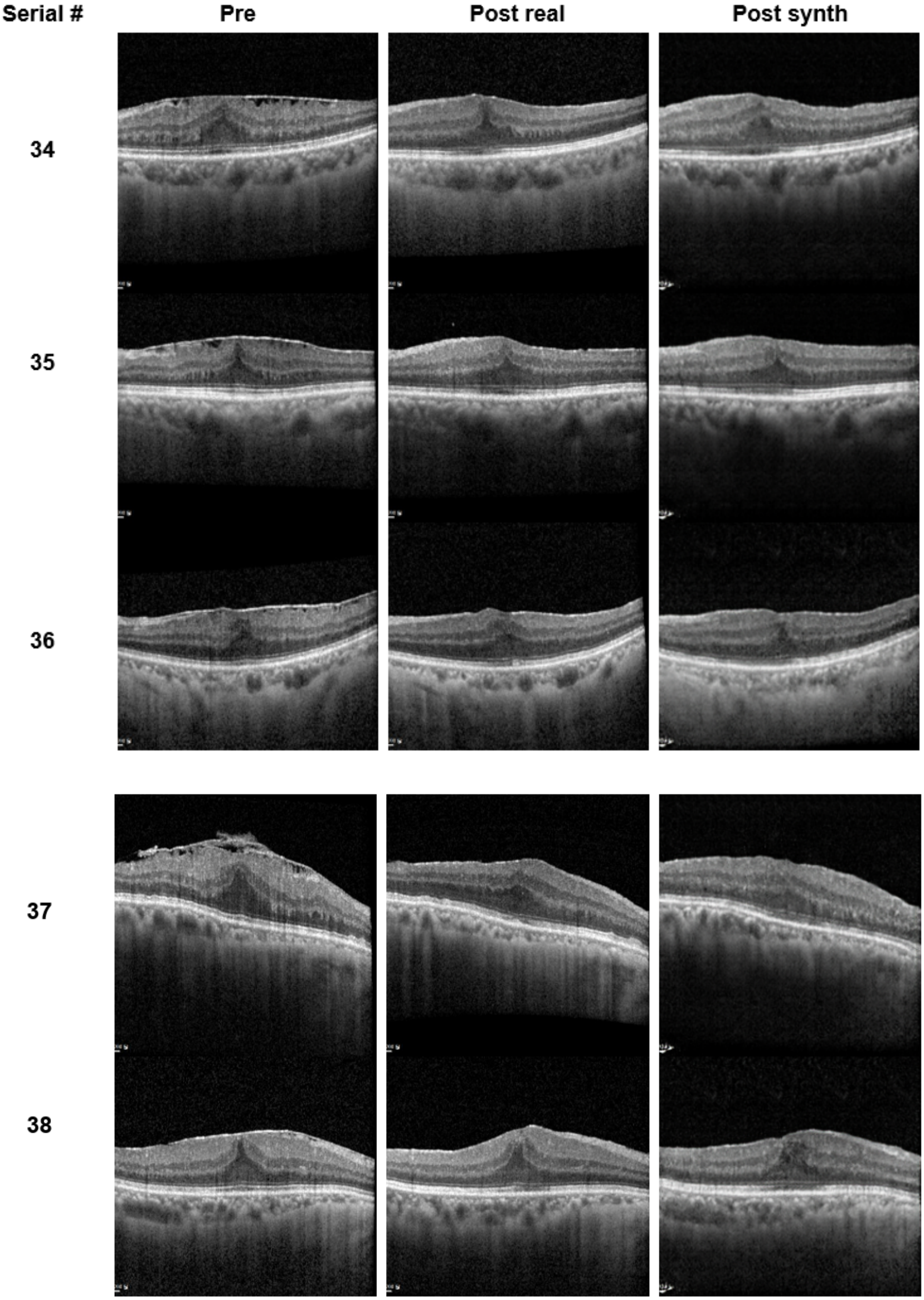
Serial #
Pre
Post real
Post synth
34
35
36
37
38

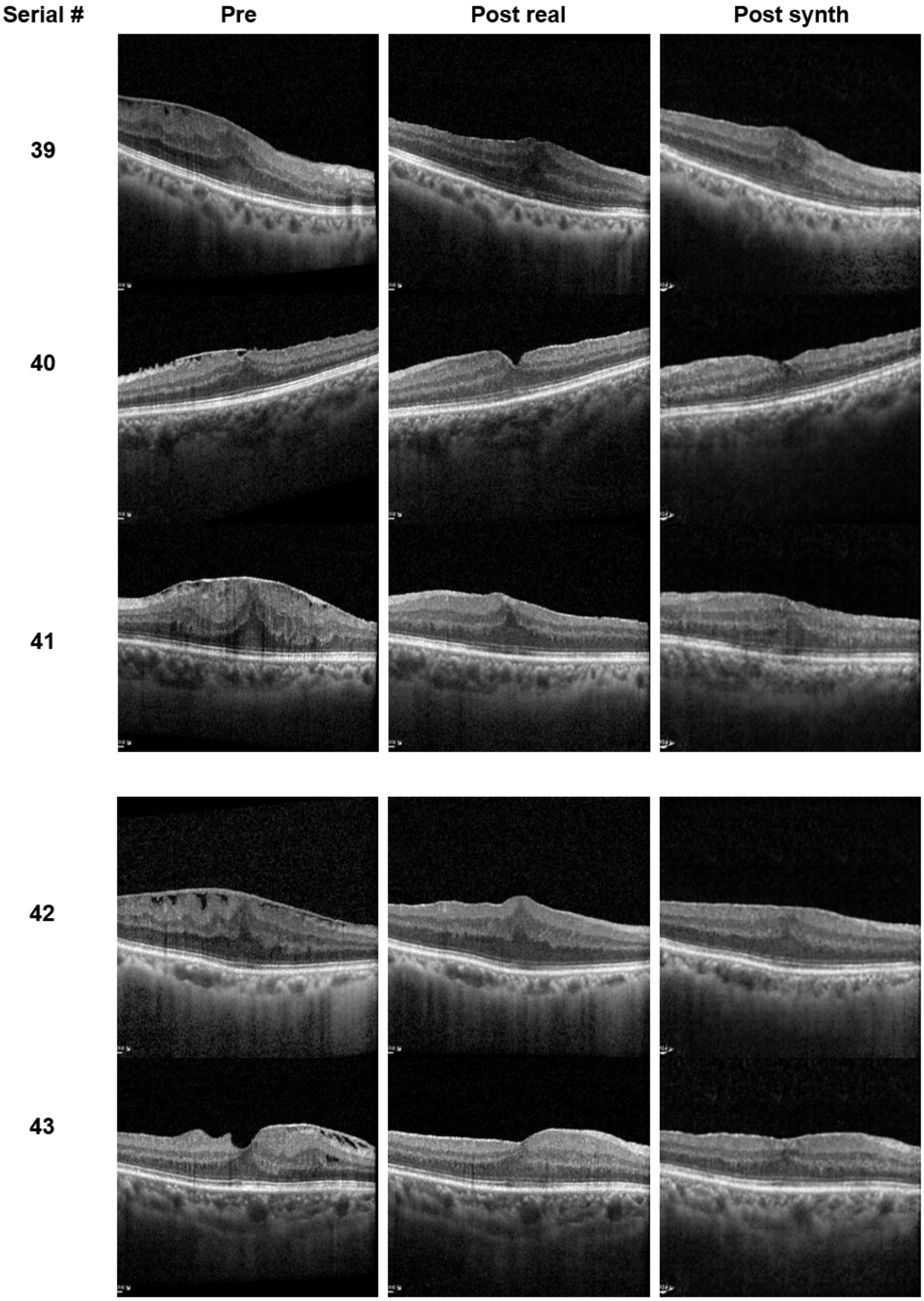
Serial #
Pre
Post real
Post synth
39
40
41
42
43

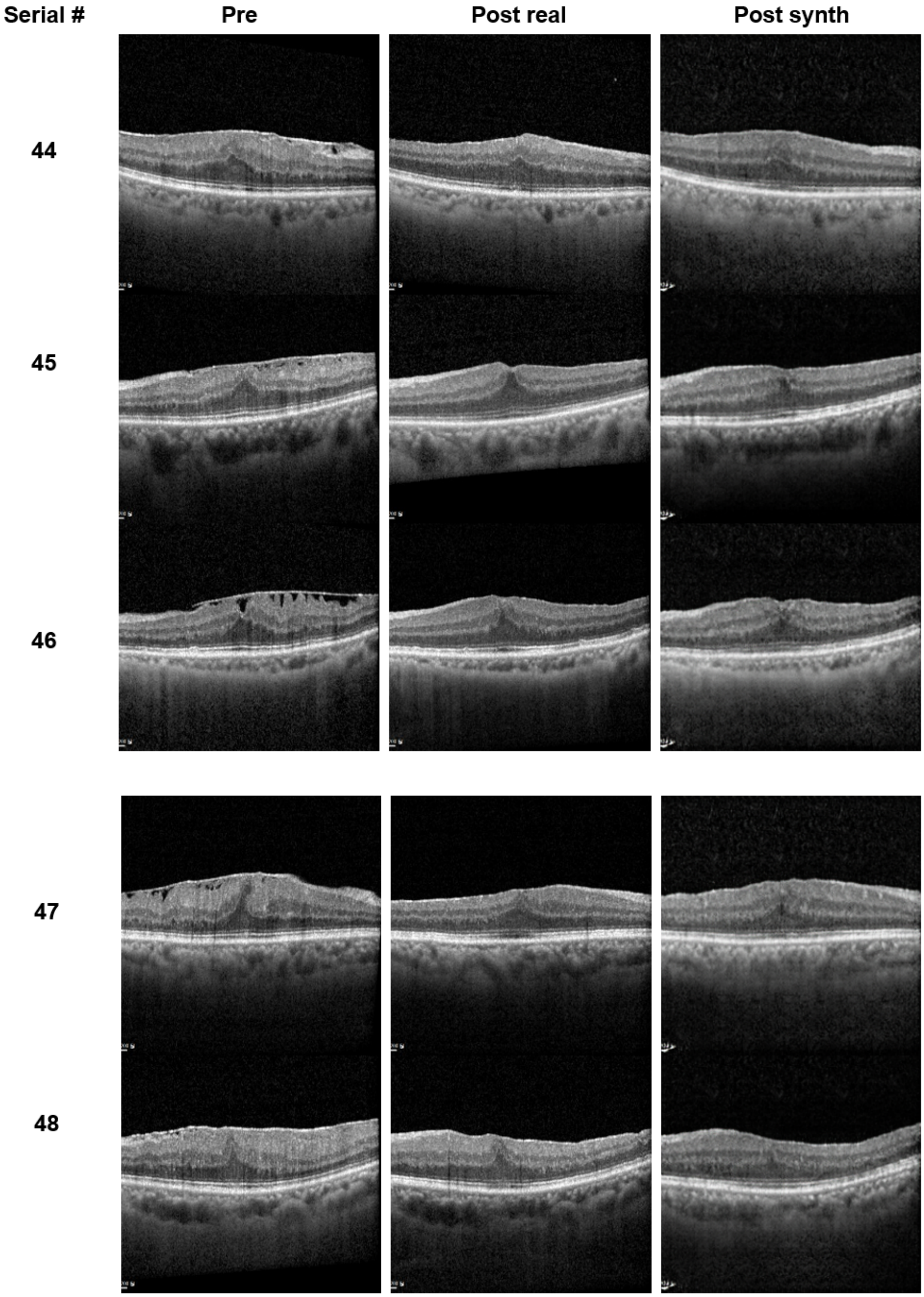
Serial #
Pre
Post real
Post synth
44
45
46
47
48

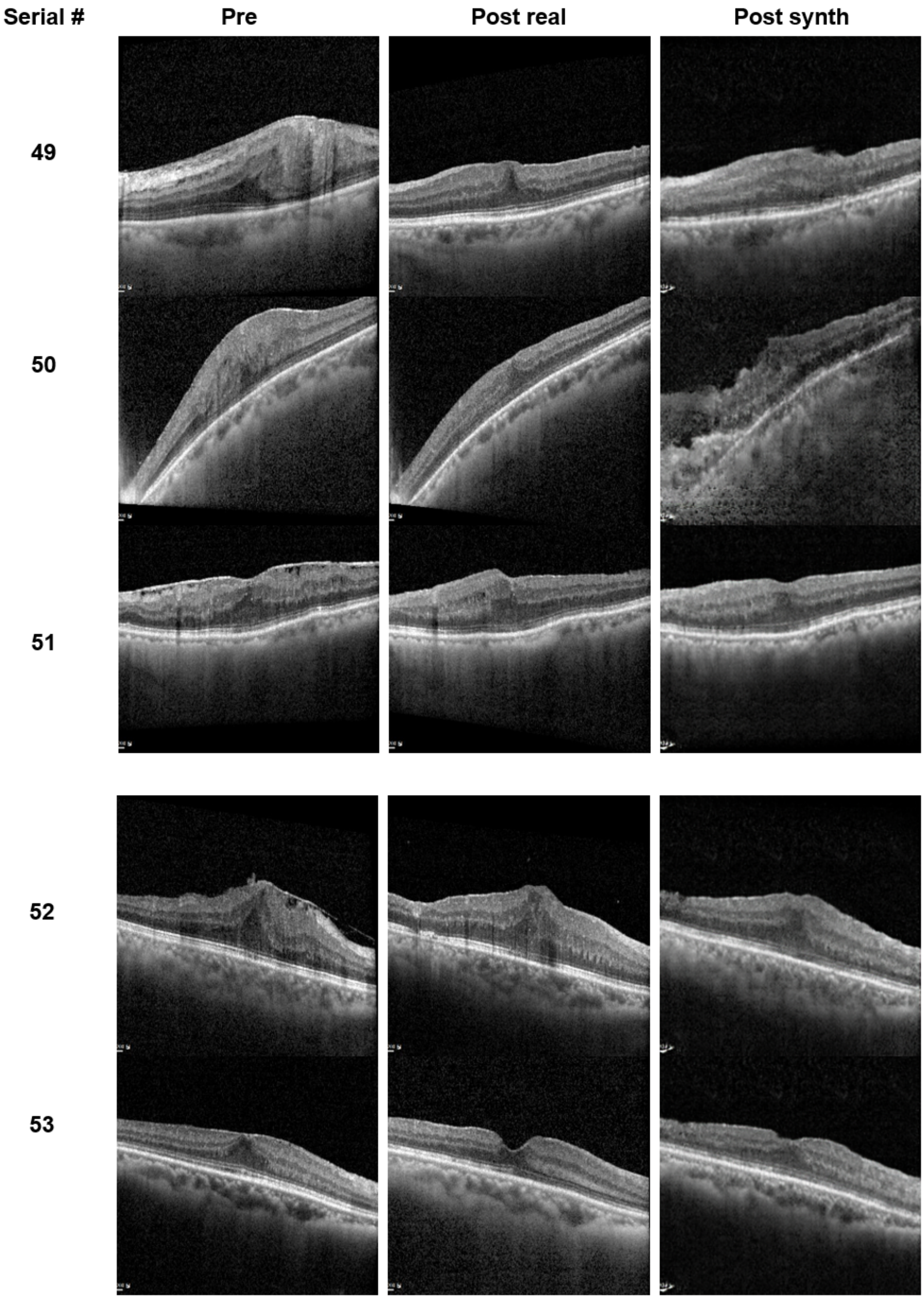
Serial #
Pre
Post real
Post synth
49
50
51
52
53

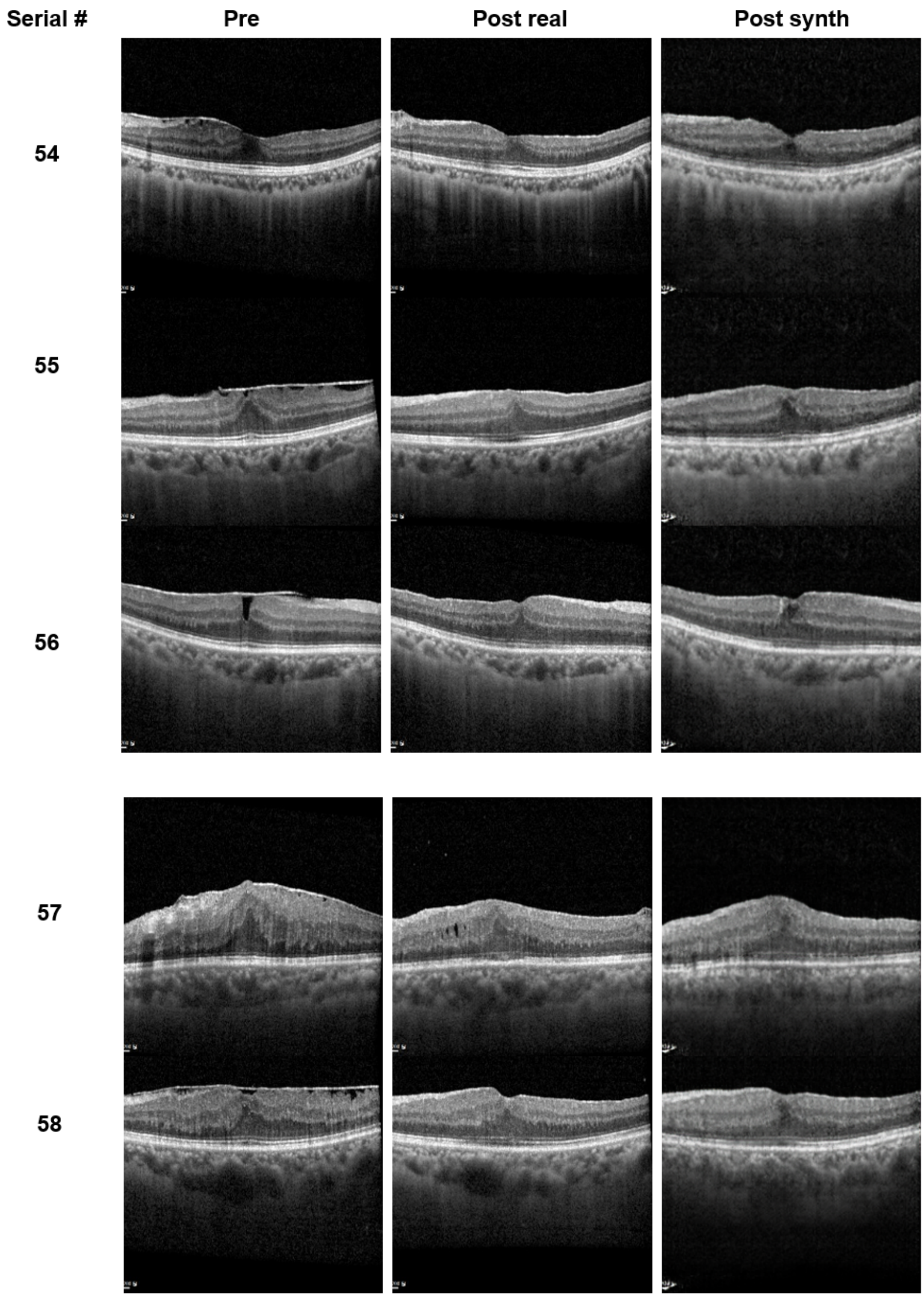
Serial #
Pre
Post real
Post synth
54
55
56
57
58

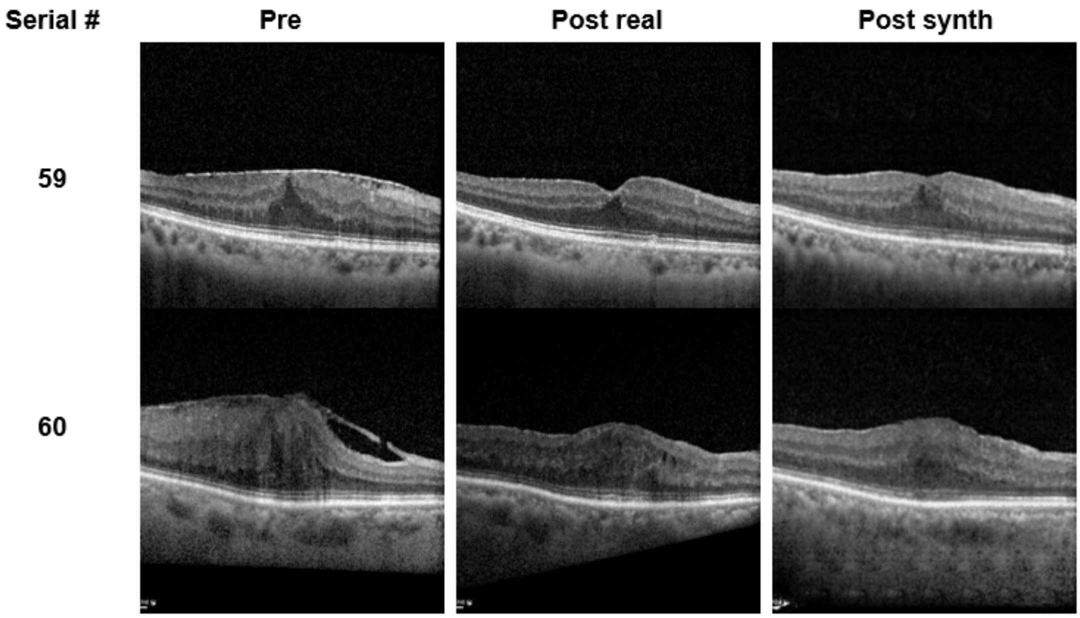

Supplementary Figure 2. Total sixty synthesized postoperative OCT images from the test datasets. Serial #: serial number, Pre: preoperative OCT, Post real: an actual postoperative OCT, Post synth: synthesized postoperative OCT
